# Identifying and addressing social determinants of health in outpatient practice: results of a program-wide survey of internal and family medicine residents

**DOI:** 10.1186/s12909-020-1931-1

**Published:** 2020-01-16

**Authors:** Lauren A. Gard, Andrew J. Cooper, Quentin Youmans, Aashish Didwania, Stephen D. Persell, Muriel Jean-Jacques, Paul Ravenna, Mita Sanghavi Goel, Matthew J. O’Brien

**Affiliations:** 1 Division of General Internal Medicine and Geriatrics, Northwestern University Feinberg School of Medicine, 750 North Lake Shore Drive, Chicago, IL USA; 2 Division of Cardiology, Northwestern University Feinberg School of Medicine, Chicago, IL USA; 3 Division of General Internal Medicine and Geriatrics; Center for Primary Care Innovation, Institute for Public Health and Medicine, Northwestern University Feinberg School of Medicine, Chicago, IL USA; 4 Department of Family and Community Medicine, Northwestern University Feinberg School of Medicine, Chicago, IL USA

**Keywords:** Social determinants of health, Graduate medical education, Primary care

## Abstract

**Background:**

Up to 60% of preventable mortality is attributable to social determinants of health (SDOH), yet training on SDOH competencies is not widely implemented in residency. The objective of this study was to assess internal and family medicine residents’ competence at identifying and addressing SDOH.

**Methods:**

Residents’ perceived competence at identifying, discussing, and addressing SDOH in outpatient settings was assessed using a single questionnaire administered in March 2017. In this cross-sectional analysis, bivariate associations of resident characteristics with the following outcomes were examined: identifying, discussing, and addressing patients’ challenges related to SDOH through referrals.

**Results:**

The survey was completed by 129 (84%) residents. Twenty residents (16%) reported an annual income of less than $50,000 during childhood. Overall, 108 residents (84%) reported previous SDOH training. Two-thirds had outpatient practices in Veterans Affairs or safety-net clinics. Thirty-nine (30%) intended to pursue a career in primary care. The following numbers of residents reported high levels of competence for performing these outcomes: identifying patients’ challenges related to SDOH: 37 (29%); discussing them with patients: 18 (14%); and addressing these challenges through referrals to internal and external resources: 13 (10%) and 11 (9%), respectively. Factors associated with higher competence included older age, lower childhood household income, prior education about SDOH, primary practice site and intention to practice primary care.

**Conclusions:**

Most residents had previous SDOH training, yet only a small proportion of residents reported being highly competent at identifying or addressing SDOH. Providing opportunities for practical training may be a key component in preparing medical residents to identify and address SDOH effectively in outpatient practice.

## Background

Social determinants of health (SDOH) are defined as “the conditions in which people are born, grow, live, work and age, and the wider set of forces and systems shaping the conditions of daily life.” [1] Prior research has demonstrated that SDOH are major drivers for diverse health outcomes and health inequities across populations [2, 3]. For example, it is estimated that up to 60% of preventable mortality is attributable to socioeconomic conditions [4]. Therefore, it is essential that health care providers learn to identify and address SDOH in order to provide comprehensive patient care and improve public health [5]. Despite the impact of SDOH on many health outcomes, most physicians do not feel confident addressing these factors in their clinical practice [6].

Undergraduate medical education on SDOH is recommended by the American Association of Medical Colleges (AAMC) and was recently required by the Liaison Committee on Medical Education (LCME) [7]. However, medical education primarily focuses on specific pathogens and diseases, individual risk factors, and medical treatments. Many have advocated for integrating social determinants of health in graduate medical education training, specifically teaching communication skills to elicit SDOH and developing skills to address related issues [5]. The Accreditation Council for Graduate Medical Education (ACGME) includes some SDOH-related competencies, such as engaging cultural and economic factors that affect patient care. However, these competencies are not widely implemented during residency, as programs attempt to develop best practices for training on this topic [8].

Primary care residency training offers a unique opportunity to learn about identifying and addressing SDOH, given these specialties’ focus on providing comprehensive care, coordinating medical and social services, and maintaining longitudinal doctor-patient relationships [9]. However, little is currently known about primary care residents’ practice related to SDOH, particularly in internal and family medicine. The objectives of this study were to assess residents’ self-reported competence performing the following functions related to SDOH in patient care: 1) identifying SDOH during outpatient encounters, 2) discussing those challenges with patients, and 3) making referrals to address SDOH both within and outside the healthcare system.

## Methods

### Study design and setting

This was a cross-sectional survey study following a purposive sampling approach. All current family medicine and internal medicine residents at Northwestern University’s Feinberg School of Medicine and the McGaw Medical Center of Northwestern University were eligible to participate in the study (*n* = 39 and 115, respectively). Survey data were collected from March 2017 to May 2017 and analysis was conducted in February to May 2018. The study and consent procedures were approved by the Northwestern University Institutional Review Board.

### Measurements

Our literature review revealed no validated questionnaires measuring medical trainees’ knowledge or practice related to the SDOH. Therefore, we developed a questionnaire for this study by adapting items from previously published instruments assessing related areas [10–14]. We followed Moore’s taxonomy of outcomes as a framework to develop questions assessing residents’ self-reported competence identifying and addressing SDOH [15]. The final questionnaire was developed through an iterative process involving a diverse team composed of residents, medical educators, health services researchers, and data analysts, who examined and revised questions to maximize content and face validity. Team members ultimately reached consensus on the final questionnaire items, which are included in the Appendix (see Appendix 1). Two residents on the study team were eligible to complete the survey because their role was limited to reading survey questions for comprehensibility and face validity.

We examined four outcomes related to residents’ perceived competence at identifying and addressing SDOH in outpatient practice. These were assessed by asking, “What is your level of competence performing the following tasks?”: 1) “identifying challenges to optimal health care that affect patients of low socioeconomic status”; 2) “discussing these challenges during your patients’ routine office visits”; 3) “referring patients to resources within Northwestern Medicine to address these challenges”; and 4) “referring patients to local community resources outside Northwestern Medicine to address these challenges.” Responses for each item were assessed on a 5-point Likert scale (novice, minimally knowledgeable, competent, highly experienced, and expert), and dichotomized into highly experienced or expert versus other. This cut-point was chosen based on feedback from senior medical educators whose goal is that residents become highly experienced or expert in this area.

### Data collection

All 154 eligible residents received an email from the research coordinator briefly describing the study and inviting them to participate with a link to the internet-based consent form and questionnaire. Participants provided informed consent via an electronic consent form and signature. Once informed consent was obtained, the participant was redirected to the online survey. Survey responses were collected and managed using Research Electronic Data Capture (REDCap) on secure servers hosted at Northwestern University Feinberg School of Medicine [16]. No data were collected from residents who declined to participate. Residents were considered non-responders if they did not respond to three reminder emails to complete the questionnaire. Those who participated received a $50.00 gift card as compensation for their time.

### Statistical analysis

Descriptive statistics were calculated to summarize the responses to all survey items and examine their distributions (Table 1). After dichotomizing the four outcomes described above, Fisher’s exact test was used to examine the bivariate association of categorical participant characteristics with the outcomes. Residents who did not respond to a question or responded “Don’t Know” were excluded from the analysis. *P*-values of 0.05 were considered significant for all statistical testing. Data were analyzed using SAS, version 9.4 (SAS Institute; Cary, NC).

**Table 1:** Characteristics of Resident Respondents (*n* = 129)

Characteristic	n (%)^a^
Specialty	
Family medicine	34 (26)
Internal medicine	95 (74)
Prior Training in SDOH	
Yes	108 (84)
No	21 (16)
Age, years	
25 to 29	74 (57)
30 to 34	47 (36)
35 to 44	8 (6)
Female sex	69 (54)
Post-Graduate Year (PGY)	
PGY-1	46 (36)
PGY-2	40 (31)
PGY-3	43 (33)
Race/ethnicity	
White or Caucasian	70 (54)
Black or African American	11 (8)
Hispanic or Latino	15 (12)
Asian or Pacific Islander	28 (22)
Other/more than 1 race	5 (4)
Household income during childhood, dollars	
≤ $50,000	20 (17)
$50,001–$100,000	34 (30)
$100,001–$200,000	30 (26)
≥ $200,001	31 (27)
Highest level of parental education	
≤ College graduate	18 (14)
Primary outpatient practice site^b^	
Academic/private practice	44 (34)
Safety-net^c^	31 (24)
Veterans Administration	54 (42)
Intention to practice primary care^d^	
Yes	39 (30)
No/Unsure	90 (70)

*SDOH* social determinants of health

^a^ The denominator for some resident characteristics does not add to 129 due to non-response for some items

^b^ Determined based on residents’ response to a question asking them to identify the outpatient setting where they see the majority of ambulatory patients

^c^ Includes federally qualified health centers and a Northwestern clinic that serves uninsured and underinsured patients

^d^ Determined based on residents’ response to a question asking whether they planned to pursue a career in primary care after completing residency

## Results

### Resident characteristics

The survey was completed by 129 residents [95 (74%) in internal medicine and 34 (26%) in family medicine], with an overall response rate of 84%. The characteristics of resident respondents are presented in Table 1. The mean age was 29.7 years, with a roughly equal distribution of respondents by post-graduate year (PGY). Over half of residents reported white race (70; 54%), and 54 (42%) reported Black, Hispanic or Asian race/ethnicity. Residents came from households with relatively high socioeconomic status, as evidenced by high levels of parental income and education. Most respondents (108; 84%) reported having received education about the SDOH before residency. Two-thirds of residents had outpatient practices that included a VA or safety-net clinic; and approximately one-third planned to pursue careers in primary care after completing residency.

Overall, less than one-third of residents reported being highly experienced or expert at identifying and addressing SDOH in outpatient practice (Table 2). The following resident characteristics were associated with one or more of the four outcomes: age, household income during childhood, prior education on the SDOH, primary clinic site, and intention to practice primary care. The oldest residents were significantly more likely than their younger counterparts to report being highly experienced or expert at discussing challenges related to SDOH and referring patients to community-based resources to address these challenges. Older residents were also more likely to report the same level of competence for the other two outcomes, with bivariate associations that approached statistical significance. Residents with the lowest household income during childhood reported greater levels of competence for all outcomes. However, only the association between childhood household income and self-reported competence referring patients to external resources was statistically significant.

**Table 2:** Bivariate Association between Resident Characteristics and Self-Reported Competence Identifying and Addressing Social Determinants of Health

Characteristic		Self-Reported Competence of Highly Experienced or Expert^a^							
	Denom. in category	Identifying Challenges^a^		Discussing Challenges^a^		Referring to Internal Resources^a^		Referring to External Resources^a^	
	n	n (%)	P-value^b^	n (%)	P-value^b^	n (%)	*P*-value^b^	n (%)	*P*-value^b^
Overall	129	37 (29)		18 (14)		13 (10)		11 (9)	
Prior Training in SDOH			0.007		0.04		1.00		0.21
Yes	108	36 (33)		18 (17)		11 (10)		11 (10)	
No	21	1 (3)		0 (0)		2 (10)		0 (0)	
Age, years			0.08		0.008		0.10		0.02
25 to 29	74	16 (22)		5 (7)		9 (12)		6 (8)	
30 to 34	47	17 (36)		10 (21)		2 (4)		2 (4)	
35 to 44	8	4 (50)		3 (38)		2 (25)		3 (38)	
Annual household income during childhood, dollars^c^			0.22		0.16		0.26		0.03
≤ $50,000	20	9 (45)		6 (30)		4 (20)		4 (20)	
$50,001–$100,000	34	11 (32)		3 (9)		3 (9)		3 (9)	
$100,001–$200,000	30	6 (20)		3 (10)		1 (3)		1 (3)	
≥$200,001	31	7 (23)		3 (10)		4 (13)		0 (0)	
Primary outpatient practice site^d^			< 0.001		< 0.001		0.09		0.001
Academic/private practice	44	9 (21)		6 (14)		2 (5)		2 (5)	
Safety-net^e^	31	18 (58)		10 (32)		6 (19)		7 (23)	
Veterans Administration	54	10 (19)		2 (4)		5 (9)		2 (4)	
Intention to practice primary care^f^			0.001		0.02		0.21		0.003
Yes	39	19 (49)		10 (26)		6 (15)		8 (21)	
No/Unsure	90	18 (20)		8 (9)		7 (8)		3 (3)	

^a^ Residents reported their competence at identifying and addressing the social determinants of health by answering: “What is your level of competence performing the following tasks? 1) identifying challenges to optimal health care that affect patients of low socioeconomic status; 2) discussing these challenges during your patients’ routine office visits; 3) referring patients to resources within Northwestern Medicine to address these challenges; and 4) referring patients to local community resources outside Northwestern Medicine to address these challenges.” Responses to these questions were answered on a 5-point Likert scale and dichotomized into Highly Experienced or Expert vs. Other

^b^ *P*-values were derived from Fisher’s exact tests examining the bivariate association between resident characteristics and self-reported competence addressing the social determinants of health

^c^ This bivariate analysis was limited to participants with non-missing values for the survey item about annual childhood household income (*n* = 115)

^d^ Determined based on residents’ response to a question asking them to identify the outpatient setting where they see the majority of ambulatory patients

^e^ Includes federally qualified health centers and a Northwestern clinic that serves uninsured and underinsured patients

^f^ Determined based on residents’ response to a question asking whether they planned to pursue a career in primary care after completing residency

Residents who had previous education on SDOH were significantly more likely to report high competence at identifying and discussing challenges related to this topic. However, prior education about SDOH was not significantly associated with making referrals to address those challenges. Residents practicing in safety-net clinics were significantly more likely to report being highly experienced or expert for all outcomes except referring patients to internal resources. The same pattern was observed for the association between intention to practice primary care and the four competency outcomes.

## Discussion

This study examined family and internal medicine residents’ perceived competence at identifying and addressing SDOH in outpatient practice and found that few residents rated themselves as being highly experienced or expert in these areas. Factors associated with higher perceived competence included age, lower household income during childhood, prior education about SDOH, primary practice site in a safety-net clinic, and intention to practice primary care. These findings have implications for residency training on SDOH, which is an emerging priority in graduate medical education.

Residents who were older and came from less affluent families reported higher levels of competence in some outcomes related to SDOH, which may suggest that residents’ lived experiences inform their practice in this area. Most residents received prior training on SDOH before residency, which was significantly associated with higher perceived competence in identifying and discussing patient challenges but not making referrals to address them. This finding may highlight a need for practical and concrete training on how to address SDOH challenges that are identified during the medical history.

The greater perceived competence among those who plan to enter primary care practice may suggest a selection effect given that this represents a common venue for addressing SDOH. Alternatively, those who plan to practice in primary care settings after residency may pursue educational experiences that build their skills in identifying and addressing SDOH. While our institution’s residency programs had few formal curricular efforts focused on this topic when the survey was administered, some residents may have attended optional lectures or pursued volunteer opportunities where they learned more about this topic than their peers.

The strongest predictor of residents’ perceived competence identifying and addressing SDOH was having their primary outpatient practice at a safety-net clinic. This likely reflects their greater experience addressing this topic while caring for socioeconomically vulnerable patients in these settings. Federally qualified health centers are required to provide comprehensive services that include multidisciplinary team members such as social workers and case managers, in addition to some wrap-around services like transportation that directly address SDOH [17]. Therefore, these clinics likely have greater resources for addressing SDOH than other primary care settings.

Most existing literature on SDOH in medical education consists of elective course descriptions with accompanying evaluations of outcomes among learners [18]. We are unaware of program-wide evaluations of residents’ competence addressing SDOH in their outpatient practice. Assessing residents’ baseline competence in this area helps determine their specific needs, which may help programs design more targeted curricular programs.

Our study has some notable limitations. It was conducted at a single institution; and therefore, the findings may not be generalizable to other internal and family medicine programs. Much curricular innovation focused on SDOH in graduate medical education has been led by pediatric residency programs [19–24]. These programs have addressed SDOH by integrating novel services in pediatric clinics, developing partnerships with community-based agencies, and teaching communication skills around this topic. We designed the current study to include residents from other primary care specialties where there has been little curricular focus on SDOH to date.

Like many similar studies, our outcomes were assessed by self-report because there are no objective tools for evaluating competencies related to SDOH. Further, because there is little consensus about what skills are required to demonstrate competence in this area, the study questionnaire asked residents to report their perceived competence without an a priori definition of each category (e.g. highly experienced or expert). Our study focuses on U.S. residency training; however, programs in low and middle income countries may have more experience training residents to identify and address SDOH. Our literature review did not identify international studies on this topic. Finally, due to the small sample size, we had limited power to conduct multivariable analyses of the factors predicting residents’ clinical competence in this area.

In conclusion, only a minority of internal medicine and family medicine residents in our cohort reported high levels of competence in identifying and addressing SDOH. While many pilot curricula on this topic have been developed in primary care residency programs, none has been widely adopted. Importantly, our findings suggest that placing residents in safety-net settings may be an effective strategy to improve their clinical practice related to SDOH without developing additional programs. Future research is needed to test this hypothesis and to examine the impact of curricular programs on objectively measured outcomes at the resident and patient level.

## Conclusion

In our survey study of internal medicine and family medicine residents, we found that most had received prior training about the social determinants of health but less than one-third of participants felt competent addressing these issues in their clinical practice. Factors associated with high perceived competence in addressing SDOH among internal and family medicine residents were: older age, lower childhood household income, prior education about SDOH, primary care practice site in a safety-net or VA clinic, and intention to practice primary care after residency. Medical educators have noted that trainees must develop effective communication skills to elicit information about social, legal or financial needs from their patients. They must also be skilled at intervening upon the SDOH by referring patients to needed resources or collaborating with other clinical professionals who can do so effectively. Our study found that residents did not feel competent discussing these issues or making appropriate referrals to address SDOH, indicating a need to develop curricula that will prepare trainees in this critical area. Future research should also develop methods for objectively assessing residents’ competence to identify and address SDOH in their clinical practice.

## Data Availability

The datasets used and/or analyzed during the current study are available from the corresponding author on reasonable request.

## Abbreviations

AAMC: American Association of Medical Colleges
ACGME: Accreditation Council for Graduate Medical Education
LCME: Liaison Committee on Medical Education
PGY: Post-graduate year
REDCap: Research Electronic Data Capture
SDOH: Social determinants of health
